# Health promotion with physiolytics: What is driving people to subscribe in a data-driven health plan

**DOI:** 10.1371/journal.pone.0231705

**Published:** 2020-04-15

**Authors:** Tobias Mettler, Jochen Wulf

**Affiliations:** 1 Swiss Graduate School of Public Administration, University of Lausanne, Lausanne, Switzerland; 2 Institute of Information Management, University of Gallen, St. Gallen, Switzerland; Universitat de Valencia, SPAIN

## Abstract

Data-driven health promotion programs and health plans try to harness the new possibilities of ubiquitous and pervasive physiolytics devices. In this paper we seek to explore what drives people to subscribe to such a data-driven health plan. Our study reveals that the decision to subscribe to a data-driven health plan is strongly influenced by the beliefs of seeing physiolytics as enabler for positive health behavior change and of perceiving health insurances as trustworthy organizations that are capable of securely and righteously manage the data collected by physiolytics.

## 1. Introduction

Health care systems all over the world are facing a time of massive societal turmoil. While public and private health institutions are confronted with constant health reforms, restructuring, and budget cuts [1], chronic diseases, such as obesity, diabetes or cardiovascular diseases, have become a major cause of global morbidity and increased public health costs [2]. Maintaining general accessibility, efficiency, and quality of health services for a rapidly aging and less healthy population has become a key challenge of this century.

In countries with market-oriented health care systems, this growth in chronic diseases has led to a continuous rise of health premiums in the past years, making insurance coverage particularly unaffordable to people with low-to-middle-income [3]. The fact that “health care is becoming a luxury good” [4] has fueled the public debate in two directions.

First, it has favored the idea that health is not a private matter anymore [5]. Given that “chronic diseases generate billions of dollars in avoidable health expenditure every year” [6], companies and governments alike have used this argument for political reasoning and actions [7], paving the way for large-scale (digital) organizational and public health prevention and promotion programs [8–10] which have fostered the belief that much disease is caused by unhealthy life choices and that there is a moral obligation to exercise healthy behavior to reduce the financial burden on society [11]. In this sense, engaging in unhealthy behavior or even worse–having a chronic disease due to years of personal neglect and misdemeanor–imposes a moral dilemma and guilt on citizens, arising from failure or incompetence to fulfil the inherent social norms.

Second, rising costs have considerably reduced the operating margins of health insurances. While it has been common to shift costs to insured patients [12], recent political unrest and social movements indicate that such a practice cannot be sustained indefinitely. Consequently, health insurances have recently started to implement and experiment with new business models which are facilitated by physiolytics devices [13–16]. Following Wilson [17] “physiolytics” can be understood as the linking wearable computing devices, such as fitness trackers, smartphones, or smartwatches, with analytics and/or algorithmic decision-making to enhance health and well-being. By constantly tracking and accumulating a huge amount of biological, physical, behavioral, or environmental data about an insured person, it is not only possible to establish syndromic surveillance across the health system for monitoring the spread and progress of certain chronic diseases [18, 19] or for providing more accurate and contextualized health advice [20, 21]. But it also provides insights into lifestyle-dependent risks which could be used by health insurances for determining individualized pricing models [22]—yet intensifying the previously described moral dilemma by rewarding the socially assimilated and punishing the incompliant [23]. This gives rise to further concerns as health insurance companies, more than ever, become gatekeepers to health data. Not surprisingly, voices are getting louder questioning if private businesses are the right outlet for and capable of securely storing petabytes of sensitive information [24]. After all, media attention and reports on data breaches and misuse of personal information have been frequent in the past years [25, 26], causing a lot of distrust among insurance takers and nourishing the idea that health data collected by physiolytics devices could be used for unintended purposes [27].

Placed in this situation of moral and economic conflict regarding the benefits and perils of physiolytics for health promotion, this paper seeks to find an answer to the question *what drives people to subscribe to a data-driven health plan*?

To adequately respond to this research question, we therefore need an appropriate contextual envelope [28]; that is a rather *market-oriented* health care system were citizens have choice options. In this paper we thus report on the attitudes and perceptions of Swiss permanent residents regarding the intentions to subscribe to data-driven health plans. We do so because people living in Switzerland are required to purchase a standard health insurance from one of the around 50 health insurance companies in the country [29]. This obligatory insurance covers the majority of standard procedures and illness, although maintaining the freedom to choose the provider one deems most suitable as well as additional insurance coverage, such as traditional medicine, dental or, as the focus of this paper, data-driven health promotion programs.

While our reported findings certainly are context-specific–as need to be since “no theory is always wrong or always right; all are more or less relevant and helpful in different situations” [30]–our paper provides evidence and reflection about general attitudes and concerns of citizens: Given that health data, most notably collected with physiolytics devices, is becoming the currency of a new, worldwide multibillion-dollar industry [31], we aim to particularly explore three substantial hypotheses that might shed some light into an individual’s decision-making regarding the subscription to a data-driven health plan. While acknowledging the so far evidenced positive effects of digital disease prevention and health promotion [32, 33], hence, we would like to adopt a critical lens and emphasize more the constraints and less the affordances of this new technology.

In what follows, the next section introduces the concept of “physiolytics” and briefly explains its relation to current digital initiatives and health promotion. Based on the review of prior studies, we then define our hypotheses and explain our research approach subsequently. We test the proposed hypotheses with a sample of 160 people, who were randomly selected to participate in our study. We end with a discussion of these results, the limitations of our work, as well as some considerations of possible future research directions.

### 2. Background

In the past decade, we have witnessed profound changes in the types of IT-reliant solutions which are available for the mass consumer. Becoming much more ubiquitous and pervasive, the use of wearable technology has increasingly spread [34] and, to a certain extent, become mainstream for many of us [35]. Under the umbrella term “physiolytics”, Wilson [17] subsumed wearable devices and associated software that is capable of monitoring and measuring many aspects of body functions and activities for the purpose of *self-tracking*. Sensors embedded in bracelets, woven into clothing, laminated onto ultrathin skin interfaces or simply built in smartphones and tablets may be used to collect blood glucose levels, body weight, physical activity, energy expended, mood, body temperature, breathing rate, blood chemistry readings, or even brain activity [36].

Producing an extremely detailed data trail of one’s health status and behavior, physiolytics has therefore become a central element in what is known as the quantified-self movement [33]. An inherent belief of members of this movement is the assumption that “data is better than gut feeling”. Accordingly, there is a proactive stance toward obtaining information (i.e. *tracking*) and acting on it (i.e. *performance enhancement*).

A myriad of different wearable devices has been developed in the past years for supporting the quantified-self ideology, ranging from activity trackers for the consumer market to sophisticated vital sign monitors used in medical contexts. Due to the broad availability of consumer health wearables (and the rather high pricing of medically certified biosensors), prior research has primarily concentrated on the study of physiolytics for private use [37–41] and less on institutionally driven health promotion or public health digital initiatives [36, 42].

However, more recently government agencies and businesses alike have increased their investments into health apps, and social media sites to facilitate access to health-related information and providing citizens or employees with the opportunity to share experiences and promote more healthy practices [36]. Yet, these measures have not always been fruitful to unfold the desired effects on health costs [43, 44]. Given the unbound success of wearables, reaching 25.1 million shipped units in the first quarter of 2018 [45], physiolytics is therefore frequently seen as new opportunity to get a grip on the explosion of health expenditure. As opposed to passive forms of information provision, the information collected by sensors may provide users with more accurate and contextualized health advice [20, 21] and, if systematically developed, allow the creation of a syndromic surveillance service for monitoring the spread and progress of certain chronic diseases or health-related risks at work [10, 46–48]. It may, to the beliefs of certain political forces, lead to a win-win situation where citizens obtain the necessary “personalized” information and motivation needed to positively support health behavior change as well as allow government agencies and other stakeholders of the health care sector–in the first instance, health providers and health insurances–to monitor, control, and possibly alter the population’s health according to new evidence from medical research and policy goals.

Since nation-wide health information infrastructures typically develop slowly and gradually [49], and contrary to the fears of political scare mongers and techno-sceptics, we are still far away from a physiolytics-based surveillance scenario. Rather, we observe today fragmented approaches and less intrusive digital initiatives from governments and NGO’s, like the “One Million/Billion Steps Challenge” by Diabetes UK [50] or the American Public Health Association [51].

On the other hand, more and more software vendors, device manufacturers, and health insurances have started to build new digital initiatives and services, which often make systematic use of the fine-granular biological, physical, behavioral, or environmental data that are collected with physiolytics devices. In Switzerland, health insurances have been perceived as “gatekeepers to health data”, still health data protection regulation has inhibited these organizations to collect highly detailed, health-specific information about their customers. This has led to recurring asymmetric information problems between health insurances, providers, and patients. Therefore, several Swiss health insurances have established data-driven health plans including some sort of physiolytics device and software for incentivizing a healthier lifestyle of their customers and, frequently operating on the margins of legality, collecting health-specific data.

## 3. Hypotheses development

### 3.1 Expected impact on health behavior change

As we presented earlier, there has been a plethora of studies reporting on the introduction of different solutions and on the potential benefits of physiolytics for health promotion [10, 46–48, 52, 53]. However, there is hardly any empirical evidence that firmly can confirm or refute the claim that physiolytics devices are actually capable of attaining a lasting health behavior change. In fact, more recent studies [e.g. 54, 55] have been pointing to a lack of longitudinal research designs that could provide the necessary evidence for these seemingly uncontested assertions in the literature.

As a consequence, there are positive as well as not so positive attitudes towards physiolytics today [16]. We thus hypothesize, in line with traditional theories of technology adoption [e.g. 56, 57], that the expected impact of a technology is an important antecedent of use. Or in other words: Do we believe that physiolytics will help us to change our behavior to a healthier lifestyle? Do we think this technology to help us to make a difference? If the answer is positive, we assume that the likelihood to subscribe to a data-driven health plan when offered by the health insurance will be much higher as opposed to the situation when we do not believe that physiolytics will support us in becoming healthier. Accordingly, we state:

**H1.** *Expected impact on health behavior change positively influences the intention to subscribe to a data-driven health plan*.

### 3.2 Perceived risk of social cheating

Due to politics and media scrutiny, personal health has gained a societal dimension. In many countries it has become a widely-held popular belief that there is a *collective moral obligation* to exercise healthy behavior to reduce the financial burden on society [58]. The subscription to a data-driven health plan is therefore not only a question of trust in the corresponding technology and health insurance, but also a question of trust in other participants and “the system”. Given that data-driven health plans frequently are coupled with bonus programs or health premium reductions [59], and at the same time, the personalization options and safeguards of many physiolytics solutions are of rudimentary nature, one could easily imagine that some people might exploit this for a personal gain (note: equip your dog with an activity tracker and let him/her run).

We define this phenomenon of participating in a data-driven health plan without the intention to actively change health behavior, respectively to scam for health premiums only, as “*social cheating*”. Following Brinkmann [60], such a behavior is not uncommon in the insurance industry and often implicates a struggle between self-interest vs. common-interest: Transferred to our scenario, we could bring to mind that when “cheaters” constantly reach the activity goals (i.e., self-interest to get health premium), while “non-cheaters” frequently miss out goals, the insurance company might either exacerbate the pay-out of premiums, set higher bars for all, or simply suspend the data-driven health plan in the long run because of economic inefficiency (i.e. common-interest to improve population’s health in order to lower overall health expenditure). In any case, the behavior of people righteously adhering to objectives of the data-driven health plan is punished or remains unrewarded. We therefore put forward that individuals with higher distrust levels in others will probably less likely subscribe to a data-driven health plan. Or in other words, we hypothesize that social cheating will negatively influence the intention to subscribe and state:

**H2.** *Perceived risk of social cheating negatively influences the intention to subscribe to a data-driven health plan*.

### 3.3 Trust in health data governance of insurances

More than in pure leisure settings, the subscription to a data-driven health plan managed by the employer or health insurance may come with severe privacy risks [37, 39, 40]. There is a constant peril that personal data that is collected with the physiolytics device may be unconsciously (and/or illegally) accessed by third parties or repurposed for other aims than health and well-being [61]. In the past years, data breaches in the health industry have grown in both size and frequency, with the largest breaches impacting as many as 80 million people [62]. This could indeed create the impression that health insurances may either not care for or not be capable of effectively organizing their health data governance. However, this is a particularly sensitive issue, given that with physiolytics devices the information collection becomes much more detailed (and therefore valuable) as before [63].

In this sense, and in line with current work from the data privacy domain [64], we hypothesize a relationship between the intention to subscribe to a data-driven health plan and the trust one has in the health insurers’ capabilities regarding health data governance. Simply put: Do we trust the health insurance to properly handle our data that is collected by physiolytics devices? We assume that higher trust levels will positively influence the likelihood to subscribe to a data-driven health plan than in the case we experience a severe distrust in the health insurance. Hence, we state that:

**H3.** *Trust in health data governance of insurances positively influences the intention to subscribe to a data-driven health plan*.

### 3.4 Moderating effects of health status

With the goal of facilitating a generalized understanding of people’s adoption behavior, the IS field has a long tradition in applying a variance [65, 66] or variables-centered [67] approach to come to relatively robust predictive and explanatory models of IT adoption [e.g. 56, 57, 68]. However, the *user* is frequently treated as relatively uniform, discrete, and homogenous mass.

Several studies have highlighted that age [69, 70] and gender [71] are important moderators when it comes to making a decision whether or not to adopt wearable devices or health apps. While acknowledging the fact that the personal health status could be an additional relevant moderator [72], there have not been many studies that specifically investigated how a person’s condition might affect the adoption behavior of physiolytics. Certainly, determining if an individual is “being healthy” is not easy, given that it is a relative concept which experts constantly redefine and reshape in view of current societal changes and new medical evidence (note: up until the end of the 50’s smoking was not considered a health risk). That is why we let a person estimate his or her perceived health status.

According to previous studies in health services research, individuals with healthy lifestyle patterns frequently perceive and act in an affirmative way towards new health initiatives [73]. We therefore assume that people with high health develop more positive associations related to the impacts of physiolytics that people, who rated their health to be poor or high. Hence, we state that:

**H4.** *Perceived health moderates the influence of the expected impact on health behavior change on the intention to subscribe to data-driven health plan*. *A poor health status increases this influence more strongly than a good health status*.

Prior research also shows that individuals with good health have less reservations to disclose personal health information than individuals with a poor health status [74]. According to Zanin [75], people experiencing poor health conditions are also more likely to develop social distrust and anticipated victimization. In this sense, we could hypothesize that people who perceived their health as good have a lesser tendency to think of social cheating and state:

**H5.** *Perceived health moderates the influence of the estimated risk of social cheating on the intention to subscribe to data-driven health plan*. *A good health status reduces this influence more strongly than a poor health status*.

Following Bansal, Zahedi [76], individuals with a poor health status are also more likely to become sensitive about privacy concerns and data breaches. According to them, this increased awareness and perception of risk, negatively influences trust in third-parties, such as in our case health insurances. Conversely, we could assume that individuals with good health might be less prone to trust issues and therefore we state:

**H6.** *Perceived health moderates the influence of trust in health data governance of insurances on the intention to subscribe to data-driven health plan*. *A good health status reduces this influence more strongly than a poor health status*.

### 4. Research method

To test the previously defined hypotheses, we conducted a survey in which Swiss residents were asked to explore their willingness and/or hesitation to subscribe to a data-driven health plan (with specific reference to and concrete examples of existing initiatives and bonus programs of Swiss health insurance companies). Ethics approval was checked by the cantonal committee before the start of the project. The initial set of survey items for measuring the predictor variables and the dependent variable was developed based on previous studies [37, 39, 77–79]. It then underwent several iterations of psychometric assessments which resulted in the re-wording or discarding of questions following the discriminant, convergent, and nomological validity of items. The resulting items out of these iterations are presented in Table 1. In order to measure the three direct predictors and the dependent variable, we used a 5-point Likert scale anchored with 5 = strongly agree and 1 = strongly disagree for measuring the level of agreement with our items. For the measurement of the moderator variable (health), we adopted an ordinal scale with the three points 1: excellent health, 2: reasonable health, 3: poor health. We further included three control variables. Age, measured on a 3-point ordinal scale with the age ranges < 25 years, 25 to 55 years, > 55 years, may influence the intention to subscribe to a data-driven health plan; as does gender, which we measure as a dummy variable (1 = female, 0 = male), because previous research suggests a moderating effect of age and gender [80]. We further included monthly income, measured on an ordinal scale with the times 1: < 3000 CHF, 2: 3000 to 6000 CHF, 3: >6000 CHF, as a control because income is considered a key confounding variable for studying technology acceptance and usage [81].

**Table 1:** Description of items, their loadings and cross-loadings.

*Construct*	*Item*	*Question*	*Mean*	*SD*	*INT*	*IMP*	*SOC*	*GOV*
Intention to subscribe to a data-driven health plan (INT)	INT1	I would like to get the opportunity to have a test run with a data-driven health plan	2.77	0.83	**0.52**	0.08	0.05	0.30
	INT2	I intend to subscribe to a data-driven health plan that provides me with a physiolytics device	2.83	0.79	**0.78**	-0.11	-0.01	-0.10
	INT3	I’m looking for a health insurance offering a data-driven health plan	2.57	0.82	**0.64**	0.12	0.00	-0.02
	INT4	I’m waiting for my health insurance to offer a data-driven health plan	2.38	0.96	**0.33**	0.19	-0.12	0.15
Expected impact on health behavior change (IMP)	IMP1	Using physiolytics will help me to accomplish my health goals	2.93	0.95	0.09	**0.75**	0.01	-0.04
	IMP2	Using physiolytics will improve the quality of my daily health	2.77	0.99	-0.07	**1.04**	-0.02	-0.09
	IMP3	Using physiolytics will positively influence my lifestyle	2.87	1.00	0.09	**0.63**	0.09	0.17
Perceived risk of social cheating (SOC)	SOC1	I distrust my health insurance to take the necessary measures to inhibit cheating	2.01	0.96	0.05	-0.03	**0.79**	-0.03
	SOC2	I fear that others will manipulate the physiolytics device	1.82	0.90	-0.03	0.03	**0.73**	-0.06
	SOC3	I fear that others will cheat during the data collection	2.03	1.02	-0.09	0.08	**0.75**	-0.06
	SOC4	I believe that others will participate for the unique purpose of obtaining a monetary gain	2.17	0.93	0.07	-0.07	**0.76**	0.04
Trust in health data governance of insurances (GOV)	GOV1	I believe that my health insurance is capable of properly handling my health data	2.97	0.98	-0.09	-0.08	0.01	**0.85**
	GOV2	I feel at ease that my health insurance is managing the data collected with my physiolytics device	2.86	0.95	0.11	-0.01	-0.09	**0.61**
	GOV3	I trust my health insurance not to repurpose the data collected with my physiolytics device	2.85	0.90	0.01	0.00	0.00	**0.90**
		*Number of factors*			*1*	*2*	*3*	*4*
		Eigenvector			4.92	1.74	**1.07**	0.62
		Velicer’s minimum average partial test			0.00	**0.78**	0.68	0.64
		RMSEA			0.19	0.15	0.09	**0.06**

Respondents for this study were recruited through social media, announcements on our website, and by engaging with people on the streets. Participation in the study was voluntary and after written consent. We obtained a sample of 160 valid responses. Out of the total sample, 55.6% were male and 44.4% female. The age of the respondents ranged from 18 to 76; 40.6% were below 25 years, 32.5% between 25 and 55, and 26.9% older than 55 years. 75.6% declared themselves to be in excellent health, 11.9% in reasonable health, and 12.5% expressed to be in rather poor health conditions. From a financial perspective, 38.8% had less than 3,000 CHF a month in disposable income, 31.9% between 3,000 and 6,000 CHF, and 29.4% a monthly budget of more than 6,000 CHF.

## 5. Results

We conducted an exploratory factor analysis with oblique rotation for measurement model assessment. Table 1 provides an overview of the operationalization of items, their loadings, and cross-loadings. The oblique-rotated items exhibit acceptable factor loadings of above 0.5 and negligible cross-loadings of below 0.3, which asserts the constructs’ discriminant validity [82, p. 649]. The only exception is INT4 with a relatively low factor loading of 0.33. However, we did not drop this item because there is a considerable difference between the factor loading and cross-loadings (of .14 and above).

While the eigenvalues-greater-than-one rule [83] favors three factors, the Velicer’s minimum average partial test [84] suggests the choice of two factors. The Root Mean Square Error of Approximation (RMSEA) drops below the 0.8 threshold, which indicates fair model fit, at four factors [85]. Because this is in line with our theoretical instrument development, we choose the four-factor solution.

Table 2 exhibits the constructs’ *α* values and correlation coefficients. We use the coefficient Cronbach’s Alpha (*α*) to determine the reliability of the operationalized constructs. According to Cortina [86] the values for *α* should be greater than 0.7 for an acceptable scale. Because all alphas are greater than 0.7, the scales have sufficient convergent validity to measure all model constructs.

**Table 2:** Construct reliability and inter-construct correlations.

*Construct*	*α*	*INT*	*IMP*	*GOV*	*SOC*	*HEA*	*GEN*	*Age*	*INC*
Intention to subscribe to a data-driven health plan (INT)	.72		.37***	.56***	-.48***	-.02	.02	.00	.03
Expected impact on health behavior change (IMP)	.86	.37***		.28***	-.19*	.03	.04	-.03	-.03
Trust in health data governance of insurances (GOV)	.83	.56***	.28***		-.42***	-.00	.01	-.01	.16*
Perceived risk of social cheating (SOC)	.85	-.48***	-.19*	-.42***		-.03	-.02	-.09	-.02
Health (HEA)		-.02	.03	-.00	-.03		.03	.09	.08
Gender (GEN)		.02	.04	.01	-.02	.03		.18*	.04
Age		.00	-.03	-.01	-.09	.09	.18*		.05
Income (INC)		.03	-.03	.16*	-.02	.08	.04	.05	

* p < .05

** *p* < .01

*** *p* < .001

Table 3 reports the results of the hierarchical regression analysis [87]. In Model 1, we only included the controls. In Model 2, we further added the three main effects. F-change is significant (0.42***); the three main effects thus contribute to explaining the intention to subscribe to a data-driven health plan. The beta value for the expected impact on health behavior change is positive and significant (0.21**), which supports H1: The expected impact on health behavior change positively influences the intention to subscribe to a data-driven health plan. The beta value for the perceived risk of social cheating is negative and significant (-0.28***), which supports H2: The perceived risk of social cheating negatively influences the intention to subscribe to a data-driven health plan. The beta value for trust in health data governance of insurances is positive and significant (0.39***), which supports H3: Trust in health data governance of insurances positively influences the intention to subscribe to a data-driven health plan. In Model 3, we further added the interaction terms of health with the three main effects. F-change is again significant (0.05*). The interaction terms thus also contribute to explaining the intention to a data-driven health plan. The beta value for the interaction term of health with the expected impact on health behavior change is negative and significant (-0.29**), which supports H4: Perceived health moderates the influence of the expected impact on health behavior change on the intention to subscribe to data-driven health plan. A poor health status weakens this influence. The beta value for the interaction term of health with the perceived risk of social cheating is negative and significant (-0.27*), which supports H5: Perceived health moderates the negative influence of the estimated risk of social cheating on the intention to subscribe to data-driven health plan. A poor health status increases the negative influence. The beta value for the interaction term of health with trust in health data governance of insurances is insignificant (-0.12). Thus, H6 is not supported.

**Table 3:** Hierarchical regression analysis.

	*Model 1 Controls*	*Model 2 Main Effects*	*Model 3 Moderation*	
*Controls*:				
Age	-.00 (-0.02)	-.01 (-0.17)	.01 (0.12)	
Gender	.02 (0.18)	.00 (0.02)	.02 (0.28)	
Income	.03 (0.43)	-.03 (-0.43)	-.04 (-0.71)	
*Predictors*:				
Expected impact on health behavior change		.21 (3.19**)	.58 (3.85***)	H1 (sup.)
Perceived risk of social cheating		-.28 (-4.04***)	.06 (0.40)	H2 (sup.)
Trust in health data governance of insurances		.39 (5.45***)	.58 (3.70***)	H3 (sup.)
Health			-.02 (-0.29)	
*Interactions*:				
Health * Expected impact on health behavior change			-.29 (-2.87**)	H4 (sup.)
Health * Perceived risk of social cheating			-.27 (-2.55*)	H5 (sup.)
Health * Trust in health data governance of insurances			-.12 (-1.68)	H6 (n.s.)
R^2^ (F)	.00 (0.07)	.42 (18.64***)	.47 (13.05***)	
ΔR^2^ (F-change)		.42 (37.16***)	.05 (3.11*)	

* p < .05

** *p* < .01

*** *p* < .001 (two-tailed);

sup. = supported; n.s. = not supported

**Fig 1:**
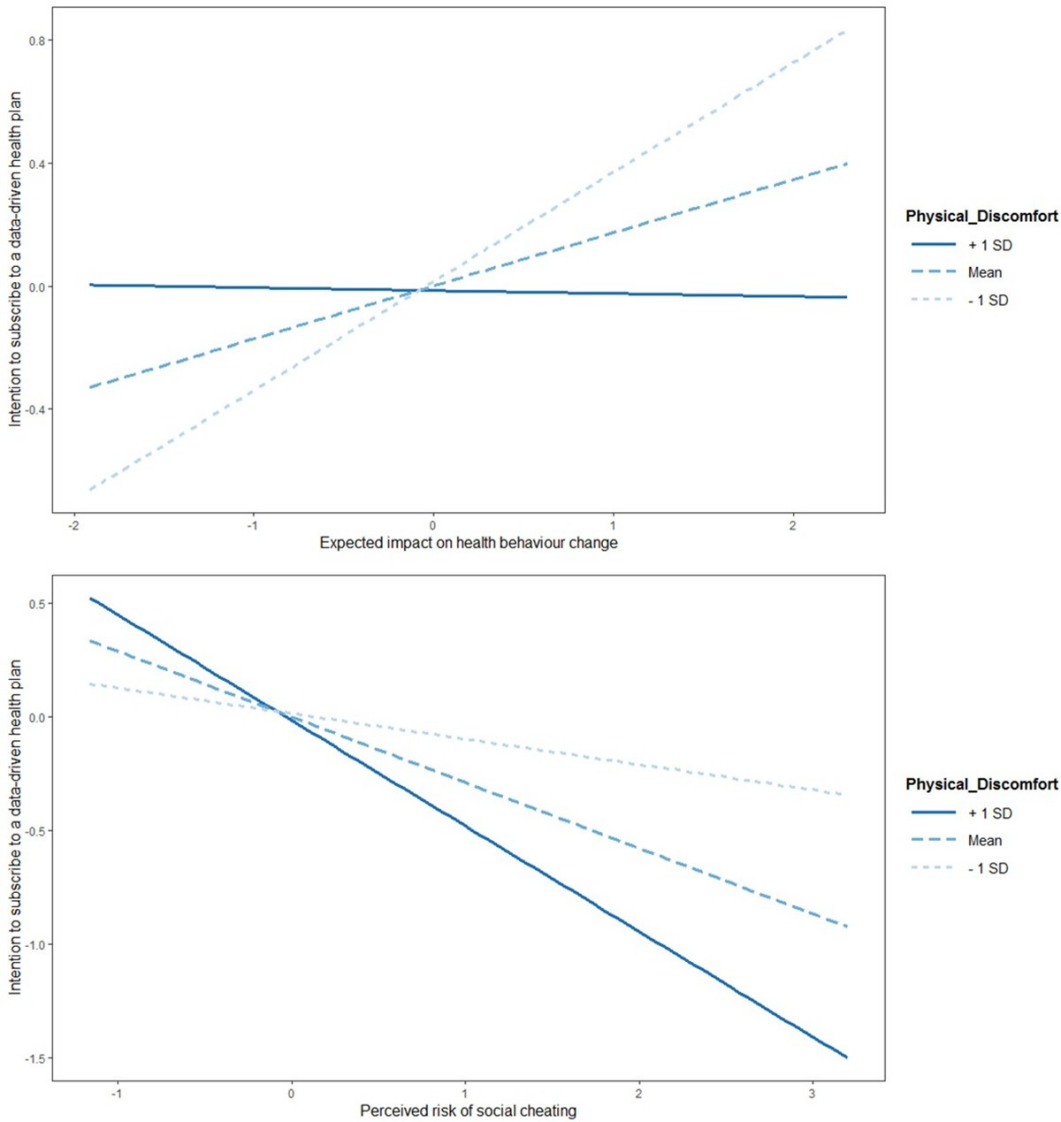
Simple slopes analysis.

Fig 1 at the left depicts the simple slopes for the influences of expected impact on health behavior change on the intention to subscribe to a data-driven health plan for three levels of health (+1 SD, mean, -1 SD). While at the -1SD-level of health a higher expected impact leads to a higher intention to subscribe, at the +1SD-level there is hardly any effect. At the right, Fig 1 depicts the simple slopes for the influences of the perceived risk of social cheating on the intention to subscribe to a data-driven health plan at the three levels of health. At the -1SD-level of health, an increase of perceived risk leads to a relatively low decrease of the intention to subscribe. At the +1SD-level, in contrast, an increase of perceived risk causes a relatively high decrease of the intention to subscribe.

## 6. Discussion

### 6.1 Expected impact of physiolytics on health behavior significantly shapes the intention to subscribe to a data-driven health plan

In line with previous research [e.g. 56, 57, 68], our results show that there is a significant correlation between the expected impact from using a physiolytics device and the intention to subscribe to a data-driven health plan. This is not so much surprising given that multiple recent studies on consumer health wearables [e.g. 69, 72, 78, 79, 88, 89] have reported on a positive relation between the expected impact (often conceptualized as perceived performance, usefulness, utility, confirmation, or value) for health behavior change and the use intention of a device. Similarly, our results suggest that individuals may be positively inclined to participate in a data-driven health plan if they genuinely believe that physiolytics can support them in achieving their health goals. There seems to be no difference for the case of institutionally driven health promotion; or in other words, if a person buys a physiolytics device on private basis or receives it from their health insurance, does not matter. What matters is the fact that this person is convinced that the device will help him/her to achieve a healthier lifestyle.

We found that this attitude towards using a physiolytics device is not dependent on age, gender, or income. Our findings rather indicate that that the relation between expected impact and use intention is moderated by a person’s health. Given that individuals with healthy lifestyle patterns perceive and act in an affirmative way towards keeping and/or improving their good health status [73], the correlation between expectations from using a physiolytics device and the intention to subscribe to a data-driven health plan is intensified. To the same extent, this is not the case with people estimating their health to be poor. According to Wikler [90] they frequently develop a tendency to self-blame or blame others for their condition and therefore, as opposed to quantified-selfers [33], have rather a passive stance toward self-tracking and self-enhancement.

### 6.2 Unfair behavior of others influences the intention to subscribe in a data-driven health plan

Our analysis reveals a significant negative correlation between perceived risk of social cheating and the intention to subscribe to data-driven health plans for high and low levels of health. We could interpret this result as an indication that individuals, in general, believe that others are capable of or willing to “trick the system” or that such eventual unfair behavior will negatively affect them on the long run. Interestingly, people with poor health experience more social distrust and anticipated victimization than people in good health conditions.

Without going into detail, we assume that possible explanations (“everybody cheats” and “the system delivers justice”) could also be culturally linked (we must, however, acknowledge that we lack empirical evidence justifying our explanation). Namely, it is not uncommon that people in market-oriented liberal welfare states (e.g. Canada, the United States or, as in our case, Switzerland) are often tied into the idea of “markets as systems that tend toward equilibrium”. It is socially acceptable (however, not always well-respected) to pursue one’s rational self-interest in order to help to ultimately establish an optimal “system”. The fact that people have the opportunity to cheat could simply be interpreted as market failure or sub-optimal state which needs to be fixed by the health insurance companies (*self-regulation hypothesis*). Accordingly, if these companies want data-driven health plans to be sustainable, they need to make sure that it becomes impossible to manipulate the data collection with physiolytics devices. If not, our study findings suggest that there will be otherwise a positive selection bias, given that physiolytics appears to be more attractive to people with good health than for people in poor health conditions. Hence, we could say that from a societal point of view we are in a paradoxical situation, as the overall idea is to motive unhealthy people to exercise more healthier lifestyle patterns, but data-driven health plans rather reaches the ones that are healthy.

### 6.3 Rightful health data governance is vital for data-driven health plans

Our study also shows that Swiss residents are particularly sensible to the topic of health data governance. This does not strike us, given that there has been constant media attention to data breaches, hacking attacks, and illegal data repurposing practices of health insurances, which culminated in a major lawsuit against one of the biggest Swiss health insurances [91]. In this sense, our study corroborates the findings of prior studies which equally proved that trust and privacy concerns significantly influence the intention to use consumer health wearables [42, 72, 77]. However, different from these studies, our conceptualization varies in that we did not refer to device manufacturers. Instead, we focused on the intermediary (i.e. provider of the health plan) and asked if they thought this organization would take health data governance seriously.

A possible way forward to expand this work could therefore be to apply a layered conceptualization of trust and privacy concerns, starting from device itself, the device manufacturer, and lastly the intermediary/provider of the data-driven health plan.

## 7. Conclusion

In this paper, we set out to investigate what drives people to subscribe in a data-driven health plan when given a choice in a market-oriented health system. Our results showed that believing in positive impacts of physiolytics and relying on one’s health insurance data governance capabilities are major antecedents for choosing a health promotion program based on some sort of physiolytics device. In view that boundaries between professional care and self-care, self-initiated health promotion, corporate and state-administered prevention programs are blurring, we would like to conclude this paper with a brief reflection about societal and research implications of a large-scale diffusion of data-driven health plans as well as point to some limitations and possible avenues for future research.

### 7.1 Societal and research implications of data-driven health plans

When health behaviors and body functions are digitized as quantifiable data, we might narrow down our understanding of health and a “healthy” lifestyle to the notion of a fully measurable and manageable phenomenon. In doing so, we might be only steps away from seeing life as perpetual merry-go-round of self-enhancement or telling others how to direct (or rearrange) one’s behavior. While we strongly favor the idea of self-management and self-responsibility in terms of personal health and well-being, with the introduction of publicly and privately administered health promotion programs, like data-driven health plans or corporate wellness programs, we need to be careful not to fall into paternalistic communication structures. Public and private organizations alike walk a fine line between human kindness and economizing health, or, as Lupton [36] constitutes “*encouragement becomes persuasion becomes coercion*”.

In this sense, we deem it important to not only investigate the adoption of wearables for leisure and personal use, as currently dominant in the literature [e.g. 72, 77, 89], but also draw the attention to possible tensions from institutionally driven digital initiatives. For instance, Hamel, Cortez [92] and Piwek, Ellis [93] point to the need of developing a regulatory framework for the collection or repurposing of data gathered with physiolytics devices. Despite national data protection laws in many countries posit that “personal health data belongs to patients”, we are still far away from this in practice. Given a certain financial motive of device manufacturers, health insurances, or employers, it is easily imaginable that the way we can access, manipulate, and use our own health data might be, willingly or unwillingly, delimited by organizations and as consequence bring us further away from the above-mentioned postulate.

Besides the question of how public and private digital health promotion programs will handle openness and access to our health data, we also deem it important to reflect about the issue of how these organizations might use self-tracking against us. Several scholars have pointed to the circumstance that health promotion, particularly in neoliberal political environments, tends to comprehend certain individuals or social groups as ignorant, morally deficient, lacking self-control, or incapable of taking appropriate responsibility about their health [36]. Accordingly, physiolytics in combination with data-driven health plans could become a subtle instrument for “nudging” or “directing” citizens to exert healthier lifestyles by identifying and communicating them the “right” priorities [94]. To a certain extent, this is a dangerous development because failure or incompetence to fulfil the inherent social norms and propagated values could evoke anxiety and guilt to some people and ultimately lead to a new source of inequality (e.g. the one’s conformant with the goals of the health insurance vs. the one’s at the bottom of the scoring list) and disease (e.g. depression, negative affective states, social withdrawal). The way how health promotion imperatives are presented and implemented in digital health initiatives represents, to our view, an additional dimension that could be worthwhile exploring.

### 7.2 Implications for practice

What can public and private organizations do in order to reduce possible tensions and inequalities? If data-driven health plans based on physiolytics indeed become the new standard model for population health management [59], we recommend intermediaries and providers of such personalized health solutions to consider the following:

- Educate subscribers about the positive (and negative!) results of ongoing studies on physiolytics. This should enable them to set a realistic frame of the technology’s affordances and constraints as well as clarify the user’s responsibilities for achieving a healthier lifestyle (physiolytics will not miraculously make someone healthier without any individual sacrifice and effort for changing unhealthy habits).
- Communicate health goals carefully. Do not paternalize. Do not try to coerce or persuade someone into data-driven health. Consider the initial health status of the subscriber in order to set realistic objectives, rewarding schemes, and the right tone for communication. The perceived health status of a person influences the way how they perceive the benefits and perils of physiolytics devices.
- Inform subscribers about implemented data governance practices, in particular, what health data will be collected by physiolytics devices, where and how long it will be stored, who will get access to it and what will be done with this data? This should not be restricted to the current situation only, but also describe prospective plans, such as the possible use of physiolytics data for personalized pricing models or other marketing purposes.
- Remind subscribers to keep the device up-to-date in order to reduce security risks. Also let them know about any potential attempts of data breaches or other threats to their privacy. This will show them that “health data governance” is not just a buzz word, but that it is a matter that is taken seriously.
- Let subscribers know the consequences of cheating and what measures are set in motion to detect and prevent data manipulation and other forms of cheating. This shows that one is aware of potential flaws in the (remuneration) system and keen on working to fix it. Hopefully, this reduces unfair behavior.

To sum, public and private organizations should foster a culture of open communication, allowing citizens to comprehend not just the benefits but also the perils of physiolytics, if they so desire.

### 7.3 Limitations and future research

Certainly, our study is not without limitations. First of all, it is important to notice that the findings presented in this paper might be biased by the study context and related contemporary events. Swiss residents have experienced rising insurance premiums for many years which might have caused a state of resignation in the population. While any attempt to lower health expenditure might be seen as good sign, a certain level of skepticism towards new approaches and ideas might remain. Moreover, recent lawsuits and court cases about mishandling and repurposing of health data, which received extensive media attention, might have raised particular alarm flags for some people. In this sense, certain responses to our questions might have suffered a negativity bias.

This deprecative perception about data-driven health plans might also have been intensified by the selection of our questions: Given that people in Switzerland are rather reserved and cautious about talking about their health and economic situation, we deliberately have chosen to focus our inquiry on a relatively small number of questions with the aim to achieve a high response rate and increase power of our study. Following Cohen [95] we assessed the statistical power of the full models (Models 2 and 3) as well as the individual variables and interaction terms. The resulting *power* values of 1 for Models 2 and 3 and the *power* values for the independent variables and interaction terms (minimum *power* value: .817) are all above Cohen’s suggested threshold of .8. In summary, these results increase our confidence in the validity of the analytical results.

Our questionnaire, in consequence, emphasized more the potential constraints and less the affordances of technology (e.g. asking about the ease of use or perceived enjoyment from using a physiolytics device), which frequently receives more attention in studies rigidly adhering to TAM, UTAUT, UTAUT2 as conceptual basis. This relatively small number of questions certainly limits our possibilities to control for confounding factors. It is therefore, for example, not possible to stipulate whether the current BMI or exercise level of a participant had an influence on the responses. Since we have chosen to let participants rate their health status, we can only make indirect claims and determine if *perceived* health (and not their *actual* level of health as defined by today’s standards) is influencing the intention to subscribe to a data-driven health plan.

A further limitation stems from our variance-based research design. As discussed by Beaudry and Pinsonneault [66], process-centric scholars quite rightly put forward that it is a common methodological problem to project future behavior by analyzing attitudes and intentions with a cross-sectional study design instead of observing and trying to comprehend actual behavior with a longitudinal perspective. We agree with the statement that actions speak louder than words, or, expressed more scientifically, correlation in some observed intentional variables is not causation for real behavior. However, as digital health promotion programs are still a quite recent phenomenon, it is both, difficult to collect behavioral data or to get access to such data from public and private entities.

Moreover, our results may be subject to common method bias, because the individual respondents provide ratings for both, the independent and the dependent variables. However, because our main findings relate to the moderation hypotheses and one cannot detect moderation effects in the presence of substantial method variance [96], we are confident that our results are valid.

There are multiple ways to expand this study. We propose, for instance, to apply a layered conceptualization of trust and privacy concerns, starting from the device itself, the device manufacturer, and the intermediary/provider of the data-driven health plan or to link our hypothesized model with extant health behavior theories in order to examine how the motivation to change behavior affects the intention to subscribe to a data-driven health plan. Given the financial pressure that is caused by constantly rising health premiums, the monetary incitement for participating in such a program might outweigh or even outreach the health-related motivation to prevent disease or improve one’s condition. In this case, the question of social cheating might possibly become more relevant. We also hope that our paper stimulates other researchers to dig deeper and explore both, the bright and dark side effects of physiolytics in public and private health promotion.

## Supporting information

S1 Dataset Complete data set for the analyses presented in this study. (CSV)
